# Research on multiple co-governance of agricultural non-point source pollution in China on the perspective of ENGOs and public participation

**DOI:** 10.1371/journal.pone.0280360

**Published:** 2023-02-09

**Authors:** Jing Tang, Shilong Li

**Affiliations:** 1 School of Management Science and Real Estate, Chongqing University, Chongqing, China; 2 Center for Construction Economics and Management, Chongqing University, Chongqing, China; Universidade Lisboa, Instituto superior Técnico, PORTUGAL

## Abstract

Effective prevention and control of agricultural non-point source pollution is a major challenge faced by the Chinese local government in the context of rural revitalization, and clarifying the game relationship between stakeholders in agricultural non-point source pollution control actions will help achieve multiple co-governance better. Accordingly, this paper discusses the interactive decision-making relationships between local government and livestock and poultry breeding enterprise (LPBE) under the participation of Environmental non-government organizations (ENGOs) and public, by constructing an evolutionary game model, as well as analyzing evolutionary cooperative stability strategies and realizing the simulation of evolution processes in different scenarios by MATLAB. The results show that government subsidy has an incentive effect on LPBE to adopt the purifying strategy, yet reduces the enthusiasm of local government for supervision. Improving the participation degree and right space of ENGOs is conducive to the realization of multiple co-governance models. Furthermore, the impact of public participation on multiple co-governance of agricultural non-point source pollution is related to the local government’s investigation rate and the public reporting fairness; strengthening the local government’s supervision capacity and improving the public reporting fairness can achieve better collaborative governance effects.

## 1. Introduction

Since the reform and opening up, China’s agricultural development has been soaring, creating a fantastic growth miracle. However, since agricultural activities are the "three highs" extensive production mode with high input, high output and high waste, the rapid growth of agricultural output value also significantly damages environmental resources [1, 2]. From the end of the last century to the present, the destruction of China’s rural ecological environment has become the focus of attention from all walks of life, among which the agricultural non-point source pollution caused by agricultural production and livestock and poultry breeding has attracted particular attention and become a bottleneck restricting the high-quality development of agriculture and rural areas [3]. The "Second National Pollution Source Census Bulletin" shows that China’s agricultural chemical oxygen demand, total nitrogen and total phosphorus emissions were 10.6 million tons, 1.41 million tons and 0.21 million tons respectively, accounting for 49.77%, 46.52% and 67.22% of the national emissions, and the chemical oxygen demand, nitrogen and phosphorus discharged by the poultry breeding industry were 10 million tons, 0.59 million tons and 0.12 million tons respectively, accounting for 93.76%, 42.14% and 56.46% of the total pollution emissions from agricultural sources. The intensive development of livestock and poultry breeding has increased greenhouse gas emissions [4]; consequently, mitigating the environmental impact of the transformation of livestock and poultry farming has become a challenge for many developing countries such as China [5] and India [6]. In fact, the emission intensity of agricultural non-point source pollution in China is still on the rise [7], which not only leads to the degradation of rural ecosystems through eroding soil, but also affects the health of citizens through water pollution and food source pollution. The continuous aggravation of agricultural non-point source pollution has significantly increased the incidence of foodborne diseases [8] and brought immeasurable economic losses [9]. Therefore, discussing how to effectively control agricultural non-point source pollution has become the focus of both political and academic circles in China.

The rural environment is a network organization formed by interweaving vertical and horizontal relationships with the participation of multiple stakeholders. Rural environmental governance is not only a technical problem but also a practical dilemma of collective action of "tragedy of the commons" under the influence of different interest demands and behavior-oriented conflicts of complex stakeholders [10]. Realizing the transformation from government "unified" supervision to social "diversified" governance is an inevitable measure for rural environmental governance [11–13]. However, in the actual pluralistic process, the "selective implementation" of policies by local government and the lack of responsibilities of local LPBEs lead to unsatisfactory governance effects. First, the local government may relax the control of agricultural non-point source pollution to obtain more local economic benefits [14]. Secondly, some local LPBEs discharge the pollutants under lax rural environmental protection supervision, resulting in more serious agricultural non-point source pollution. Under such conditions, it is difficult to achieve the best effect of pollution control only by the local government’s internal governance, and the external role of ENGOs and the public in agricultural non-point source pollution governance cannot be ignored. Relevant studies have shown that public participation has gradually played an essential role in environmental governance in China [15]. At the same time, ENGOs can improve the quality and enthusiasm of public participation in environmental governance [16, 17]. The high-quality green development of China and other developing countries is inseparable from public participation [18] and the assistance of ENGOs [19]. Therefore, establishing an endogenous mechanism to encourage local government, LPBE, the public and ENGOs to form a multiple co-governance model for agricultural non-point source pollution governance is a crucial measure to break through the current predicament.

In fact, many scholars have explored the endogenous mechanism to break through the dilemma of agricultural non-point source pollution control by adopting the game theory, which could describe stakeholders’ interactions and behavior decisions [20, 21]. Therefore, this paper constructs an evolutionary game model of local government and LPBE in the multiple co-governance of agricultural non-point source pollution. In studying how to give full play to the role of ENGOs and public participation in the multiple co-governance, in addition to considering the respective levels of participation of ENGOs and the public, two relevant parameters (the ENGOs’ right space and the public reporting fairness) are introduced. Based on the model, the cooperative stability evolution processes of these two groups’ behavior are analyzed. Furthermore, the impact of the relevant parameters on the stability of the game system is explored through numerical simulations, leading to relevant conclusions and policy advice. The innovation of this study mainly lies in two aspects: the first is to reject the assumption of “welfare government” and consider that local government has the characteristic of maximizing their own interests, then explore the different impacts of subsidy mechanisms on local government and LPBE; the second is to deeply analyze the strategic influence of ENGOs and public participation on both sides of the game, which provide a theoretical basis for effectively playing the role of ENGOs and public in the multiple co-governance of agricultural non-point source pollution.

## 2. Literature review

Various factors are causing agricultural non-point source pollution, among which livestock and poultry breeding pollution is the primary source of pollution [22–24]. With economic development and technological progress, the livestock and poultry breeding industry is developing in the direction of scale, modernization and intensification. Effective governance of environmental pollution caused by livestock and poultry manure emissions has become a major challenge in rural environmental governance in the new era. [5].

Local government can mitigate agricultural non-point source pollution through ecological compensation, price subsidies, technical guidance and other related initiatives [25–27]. However, problems such as "pollution while governing" and lack of local government supervision inevitably arise in the course of policy implementation. In this regard, public goods theory and externality theory have made a profound explanation: the rural ecological environment is a public resource, which determines that the environmental consumption behavior of LPBE (such as livestock manure discharge) has significant negative externalities and pollution spillover effects, thus causing a "tragedy of the commons" [28], while the behavior of agricultural non-point source pollution control has significant positive externalities, thus giving rise to the problem of "free-riding" and ultimately leading to the "prisoner’s dilemma" [29]. This shows that the problem of agricultural non-point source pollution governance is not only a technical problem, but also a real dilemma caused by the conflicting interests and behavioral orientations of complex stakeholders [30]. Therefore, classical game theory has been widely used to reveal the behaviors and decisions of multiple stakeholders in agricultural non-point source pollution [31, 32]. Adopting a multiple co-governance model involving government, enterprises, farmers and the public has become a major trend in related research [20, 33, 34]. However, most of these researches are based on traditional game theory that assumes players to be entirely rational, while, in reality, players operate within an environment of almost bounded rationality. Thus, evolutionary game theory based on limited rationality and group behavior analysis has been increasingly used to reveal the complex subject interactions and behaviors in rural environmental governance problems [35–37]. Xu et al. analyzed the governance of rural water environment using the trilateral evolutionary game, and concluded that as long as the government and enterprises take co-governance to protect the interests of farmers, it would be conducive to improving the rural water environment [10]. Cui et al., constructed an evolutionary game model between the government and farmers, farmers and enterprises, found that slashing green production costs and government regulation costs were crucial for the best stable strategy [38]. L. Xu et al. discussed the interactive decision-making relationships between new agricultural operators and traditional farmers under the guidance of local government by constructing a trilateral evolutionary game model. They suggested that it should be necessary to reduce their costs, improve incentives, and increase the common interests among groups [39]. Feng et al. analyzed the cooperation strategy of rural water environment governance PPP projects from the evolutionary game perspective, and elaborated the dynamic process between company and farmers [40].

In summary, evolutionary game theory has strong applicability to the research of agricultural production and agricultural non-point source pollution, but the interaction and bounded rationality of stakeholders should be fully considered. However, previous studies have only taken the interests of local government, farmers and agribusinesses into account, ignoring the interaction among LPBE, ENGOs and public participation for multiple co-governance. In fact, public participation has a strong influence on the strategic choices of stakeholders in environmental issues [41, 42]. As such, this paper takes local government and LPBE as the main decision-making bodies, and constructs an evolutionary game model for the collaborative governance of agricultural non-point source pollution based on the participation of the public and ENGOs, Compared with previous studies, the model in this study fully considers the limited rationality and dynamic decision-making process in which stakeholders learn and influence each other, and also pays full attention to the impact of ENGOs and public participation on the game system.

## 3. Model construction and analysis

### 3.1. Problem Description and Assumptions

**Fig 1:**
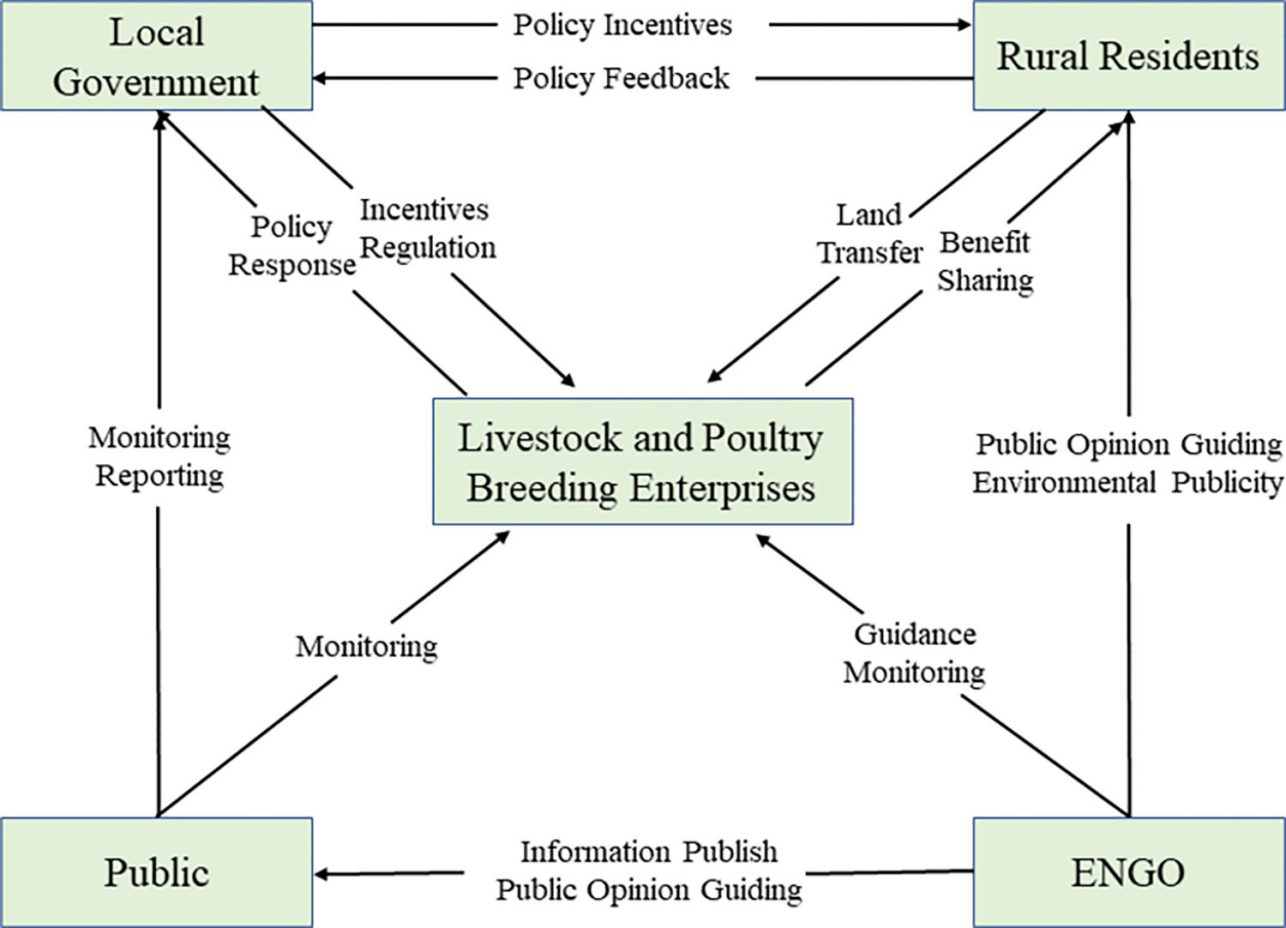
Multiple co-governance paths for agricultural non-point source pollution.

As a public and complex issue [43, 44], agricultural non-point source pollution governance is not only the responsibility of local government, but also the obligation of local LPBE. Therefore, this paper argues that the multiple co-governance of agricultural non-point source pollution within a region involves two limited rational subjects, i.e. the local government and LPBE, which are related in a way that involves both competing interests and mutual cooperation. As shown in Fig 1, the local government, as the implementer of central policies and the guide of pollution control actions, need to promote collaborative pollution control by various subjects through policy incentives, policy guidance and regulatory penalties; LPBE, as land contractors and destroyers of the rural environment, have an obligation to participate in agricultural non-point source pollution control by purifying emissions and responding to policies; other stakeholders such as ENGOs and the public can form a push-back mechanism through green production supervision and other means to control pollution at source. Through the above analysis, this paper argues that the behavioral strategies of the local government in the game process include two types, i.e. adopting “positive supervision” or “negative supervision”. And the behavioral strategies of LPBE also include two types, i.e. adopting “direct blowdown” or “purifying blowdown”.

Combined with the actual situation of agricultural non-point source pollution governance, some assumptions are presented as follows:

1. In agricultural non-point source pollution multiple co-governance, the probability of local government adopting positive supervision is *x* (*0≤x≤1*). the government will spend a fixed supervision cost *C*_0_, and ENGOs can play their role in the governance process to help the government reduce supervision costs, i.e. *C* = *C*_0_+(1−*Pω*)/*K*. Simultaneously, the government can obtain benefits *s*, which means the improvement of the rural environment and government credibility.
2. The probability that the government adopts negative supervision is 1−*x* in the governance process. At this point, although the local government needn’t to pay supervision cost, it will suffer a loss *qn* if LPBE’s irregularities are exposed, indicating the loss of credibility.
3. In the process of multiple co-governance, the probability of LPBE taking purifying blowdown strategy is *y* (0≤y≤1), at which point a certain cost *C*_*e*_ needs to be paid for the purchase of cleaning devices, etc. If the local government adopts positive supervision strategy at this time, LPBE will receive the purifying subsidy *R*_*e*_ from the government.
4. The probability of LPBE taking direct blowdown strategy is *1-y*. At this time, it will obtain an additional production income *d*, but cause a certain environmental loss *V*_*e*_. When t investigated by the local government, LPBE will be fined *F*.
5. When the public perceives that LPBE have violated the regulations, they can report it to the local government through self-media or official channels, at which point the probability that LPBE will be investigated by the government is *θ* = *λ*+(1−*λ*)*q*. Nevertheless, considering that the information reported by the public may not be completely accurate, government departments will suffer losses (1−*α*)*qn* and LPBE will suffer losses (1−*α*)*qφ* when the public reports are distorted.
6. The parameters of the game model: Under each game strategy combination, according to the cost, income, and loss of the government and LPBE, the parameter settings are shown in Table 1.

**Table 1:** Related parameters and definition.

Parameter	Definition
*x*	Probability of positive supervision of the local government, 0≤x≤1
*y*	Probability of LPBE adopting purifying blowdown, 0≤y≤1
*p*	Participation level of ENGOs, 0≤p≤1
*ω*	Right space of ENGOs, 0≤ω≤1
*k*	Local government’s supervision capacity
*q*	Public participation degree, 0≤q≤1
*α*	Public reporting fairness degree, 0≤α≤1
*φ*	Losses of LPBE due to public reporting distortion
*n*	Losses of government caused by the exposure of LPBE’s pollution
*s*	Government positive supervision benefits
*C*_0_	Fixed costs of government supervision
*C*	total costs of government supervision
*R*_*e*_	Subsidies for LPBE purifying blowdown
*C*_*e*_	costs of LPBE when purifying blowdown
*F*	Penalties for LPBE direct blowdown when being investigated
*d*	Additional benefits from direct blowdown of LPBE
*V*_*e*_	Environmental damage caused by LPBE’s direct blowdown
*θ*	probability of government investigation into the illegal blowdown behavior
λ	Local government’s own investigation rate

### 3.2. model analysis

Based on the model assumptions and the game parameters shown in Table 1, the benefits and payments of the two-party game subjects: the local government and LPBE, are shown in Table 2.

**Table 2:** Game payment matrix of government and tenants.

Players		LPBE	
		Direct blowdown (y)	Purifying blowdown (1-y)
**Local Government**	Positive supervision	*s*−*c*−(1−*α*)*qn*−*R*_*e*_,	*s*−*c*−*V*_*e*_,
	(x)	*R*_*e*_−*C*_*e*_−(1−*α*)*qφ*	*d*−*θF*
	Negative supervision	−(1−*α*)*qn*,	−*V*_*e*_−*qn*,
	(1-x)	−*C*_*e*_−(1−*α*)*qφ*	*d*−*qF*

Through the above game payment matrix, it can be concluded that the expected benefits under the strategy of positive and negative supervision behavior of local government in the process of agricultural non-point source pollution control are:

$$F_{x}=y(s-c-(1-\alpha )qn+m-R_{e})+(1-y)(s-c-V_{e})$$ (1)

$$F_{1-x}=y(-(1-\alpha )qn)+(1-y)(-V_{e}-qn)$$ (2)

The average benefits of local government in the pollution control process are:

$$\bar{F}=xF_{x}+(1-x)F_{1-x}$$ (3)

In the same way, the expected benefits for LPBE under the strategy of adopting purifying blowdown and direct blowdown behavior in the process of agricultural non-point source pollution governance are as follows:

$$G_{y}=x(R_{e}-C_{e}-(1-\alpha )q\varphi )+(1-x)({-C}_{e}-(1-\alpha )q\varphi )$$ (4)

$$G_{1-y}=x(d-\theta F)+(1-x)(d-qF)$$ (5)

The average benefits of LPBE in the pollution control process are:

$$\bar{G}=yG_{x}+(1-y)G_{1-x}$$ (6)

It can be obtained that the replication dynamic equations for the synergistic governance of agricultural non-point source pollution by local government and LPBE are as follows:

$$F(x,y)=\frac{dx}{dt}=x(1-x)(F_{x}-F_{1-x})=x(1-x)(y(-R_{e}-qn)+s-c+qn)$$ (7)

$$G(x,y)=\frac{dx}{dy}=y(1-y)(G_{y}-G_{1-y})=y(1-y)({-C}_{e}-(1-\alpha )q\varphi -d+qF+x(R_{e}+\theta F-qF))$$ (8)

Let dx/dt = 0 and dx/dy = 0, the five local equilibrium points of the evolutionary game system can be obtained as (0,0), (0,1), (1,0), (1,1), (x*, y*).


$$x^{*}=\frac{C_{e}-qF+d+(1-\alpha )q\varphi }{R_{e}-qF+\theta F}$$ (9)

$$y^{*}=\frac{c-s-qn}{-R_{e}-qn}$$ (10)

### 3.3. Equilibrium and stability analysis

The various equilibria points obtained above are not necessarily evolutionarily stable strategies for the game system. In this paper, the local stability of the equilibrium point of the evolutionary game system is judged by the Jacobian matrix composed of the partial derivatives of the dynamic equations, and the Jacobian matrix for this system is Eq (11).


$$J=[\begin{array}{cc} \frac{\partial F(x,y)}{\partial x} & \frac{\partial F(x,y)}{\partial y}\\ \frac{\partial G(x,y)}{\partial x} & \frac{\partial G(x,y)}{\partial y} \end{array}]=[\begin{array}{cc} a_{11} & a_{12}\\ a_{21} & a_{22} \end{array}]$$ (11)

$$a_{11}=\frac{\partial F(x,y)}{\partial x}=(1-2x)(s-c+qn+y(-R_{e}-qn))$$ (12)

$$a_{12}=\frac{\partial F(x,y)}{\partial y}=x(1-x)(-R_{e}-qn)$$ (13)

$$a_{21}=\frac{\partial G(x,y)}{\partial x}=y(1-y)(R_{e}-(\theta -q)F)$$ (14)

$$a_{22}=\frac{\partial G(x,y)}{\partial y}=(1-2y)({-C}_{e}-(1-\alpha )q\varphi -d+qF+x(R_{e}+\theta F-qF))$$ (15)

Through calculation, the value of the game system at the five local equilibrium points can be obtained, as shown in Table 3.

**Table 3:** Local equilibrium point value table.

Equilibrium point	*a*_11_	*a*_12_	*a*_21_	*a*_22_
(0,0)	*s*−*c*+*qn*	0	0	${-C}_{e}-(1-\alpha )q\varphi -d+qF$
(0,1)	*s*−*c*−*R*_*e*_	0	0	${-(-C}_{e}-(1-\alpha )q\varphi -d+qF)$
(1,0)	−(*s*−*c*+*qn*)	0	0	${-C}_{e}-(1-\alpha )q\varphi -d+R_{e}+\theta F$
(1,1)	*c*−*s+R*_*e*_	0	0	$C_{e}+(1-\alpha )q\varphi +d-R_{e}-\theta F$
(x*,y*)	0	A	B	0


$$A=\frac{\left(R_{e}-C_{e}-d-(1-\alpha )q\varphi +\theta F)(C_{e}-qF+d+(1-\alpha )q\varphi )(-R_{e}-qn\right)}{{\left(R_{e}-qF+\theta F\right)}^{2}}$$ (16)

$$B=\frac{\left(s-c-R_{e})(c-s-qn)(R_{e}-(\theta -q)F\right)}{{\left(R_{e}+qn\right)}^{2}}$$ (17)

If the Jacobian matrix determinant value (*a*_11_*a*_22_−*a*_12_*a*_21_) is greater than zero and the determinant trace value (*a*_11_+*a*_22_) is less than zero, the evolutionary game system has an evolutionary stability strategy (ESS). It is necessary to comprehensively consider the influence of the parameters like p, ω, q, α and *R*_*e*_ on the stable evolution state of the system. And it is difficult to determine the threshold space corresponding to each parameter, so two reference values *s*−*c*_0_ and *R*_2_ are selected, without affecting the purpose of this study, as the judgment intermediary for analyzing the impact of parameter value range change on system stability, which can be divided into the following five situations:

1. When $s-c_{0}<\frac{1-P\omega }{K}+qn$ and *d*>*qF*−*C*_*e*_−(1−*α*)*qφ*, the ESS is (0,0), that is, after continuous selection and evolution, the system finally forms a stable state in which both government’s positive supervision probability and LPBE’s purifying blowdown probability tends to 0.
2. When $s-c_{0}<\frac{1-P\omega }{K}+R_{e}$ and <*qF*−*C*_*e*_−(1−*α*)*qφ*, the ESS is (0,1), indicating the system finally forms a stable state in which government’s positive supervision probability tends to 0 and LPBE’s purifying blowdown probability tends to 1.
3. When $s-c_{0}>\frac{1-P\omega }{K}$ and *d*>−*C*_*e*_−(1−*α*)*qφ*+*R*_*e*_+*θF*, the ESS is (1,0), showing that the system finally forms a stable state in which government’s positive supervision probability tends to 1 and LPBE’s purifying blowdown probability tends to 0.
4. When $s-c_{0}>\frac{P\omega }{K}$ and *d*<−*C*_*e*_−(1−*α*)*qφ*+*R*_*e*_+*θF*, the ESS is (1,1), suggesting that the system finally forms a stable state in which both government’s positive supervision probability and LPBE’s purifying blowdown probability tends to 1.
5. When $\frac{P\omega }{K}+R_{e}<s-c_{0}<\frac{1-P\omega }{K}$ and $qF{-C}_{e}-(1-\alpha )q\varphi <d<{-C}_{e}-(1-\alpha )q\varphi +R_{e}+\theta F$, there is no ESS.

## 4. Evolutionary game analysis

In light of the above stability analysis, it is observed that the evolution of the stable state of the system depends on the relative magnitude relationship between the influence parameters *s*−*c*_0_ and *d*. Where, *s*−*c*_0_ represents the difference between the benefits gained and the fixed cost required when the local government adopts positive supervision behavior strategy, i.e. the maximum benefits gained without considering the ENGOs and public participation in the governance process; *d* represents the additional benefits that LPBE can obtain if they adopt direct blowdown strategy, i.e. the opportunity benefits obtained without considering the ENGOs and public participation. From this, it can be seen that these two values are stable in the virtual environment of agricultural non-point source pollution multi-governance, independent of the ENGOs and public participation. Therefore, the variation of the system stability equilibrium point within the range of different parameter values can be derived from a comprehensive analysis of the ENGOs participation level p, the right space ω, the public participation degree q, the public reporting fairness α and the purifying subsidy *R*_*e*_.

In order to more clearly understand the impact of the related parameters on the effectiveness of agricultural non-point source pollution multi-governance, this paper analyzes the general condition for the evolution of the system towards the (1,1) point, i.e. analyzes the situation when each parameter of the system satisfies the condition (18).


$$\{\begin{array}{c} s-c_{0}>R_{e}+\frac{1-P\omega }{K}\\ d<{-C}_{e}-(1-\alpha )q\varphi +R_{e}+(\lambda +(1-\lambda )q)F \end{array}$$ (18)

On the premise of not affecting the conclusion of the analysis, the following part will also take *s*−*c*_0_ and *d* as reference values to analyze how to set parameters, i.e. by solving the system of Eq (19) to discuss the effect of parameter changes on the function values, which leads to more general conclusions.


$$\{\begin{array}{c} minF(p,\omega )=R_{e}+\frac{1-P\omega }{K}\\ maxM(q,\alpha )={-C}_{e}-(1-\alpha )q\varphi +R_{e}+(\lambda +(1-\lambda )q)F \end{array}$$ (19)

### 4.1. Analysis of government subsidy behavior

In order to investigate the influence of government net pollution subsidies on the effect of agricultural surface source pollution control, a comprehensive analysis of the function *F*(*p*, *ω*) and the function *M*(*q*, *α*) is needed. It can be learned that both are incremental functions of the purifying subsidy *R*_*e*_, i.e. increasing the amount of purifying subsidy will increase the value of both the function *F*(*p*, *ω*) and *M*(*q*, *α*). Therefore, in order to make the system evolve better towards point (1,1), the purifying subsidy from the local government to LPBE should be controlled under the following conditions:

$$d{+C}_{e}+(1-\alpha )q\varphi -\theta F<R_{e}<s-c_{0}-\frac{1-P\omega }{K}$$ (20)

By analyzing the above equation, we can see that increasing the purifying subsidy *R*_*e*_ will increase the incentive for LPBE to adopt purifying blowdown strategy, but reduce the likelihood that the local government adopts positive supervision strategy. Conversely, reducing the purifying subsidy *R*_*e*_ will decrease the incentive for LPBE to adopt purifying blowdown strategy, yet improve the likelihood that local government adopts positive supervision strategy. Therefore, when the benefits of local government’s positive supervision are high, the purifying subsidy for LPBE can be increased appropriately, so as to increase their motivation to take the initiative to clean pollution.

### 4.2. Analysis of ENGOs participation

In order to explore the influence of ENGOs participation on the effectiveness of agricultural non-point source pollution governance, let the function *F*(*p*, *ω*) be derived from the participation degree *p* and the right space *ω* respectively. The following two equations can be obtained:

$$\frac{\partial F(p,\omega )}{\partial p}=-\frac{\omega }{K}<0$$ (21)

$$\frac{\partial F(p,\omega )}{\partial p}=-\frac{p}{K}<0$$ (22)

Eqs (19) and (20) show that the function *F*(*p*, *ω*) is a decreasing function of both ENGOs participation level *p* and ENGOs right space *ω*, i.e. increasing the participation level and the right space of ENGOs can reduce the value of the function *F*(*p*, *ω*). Therefore, in order to better satisfy the range of parameters for the evolution of the system towards the (1,1) point, it is necessary to increase the enthusiasm of ENGOs to participate in pollution governance as well as to give ENGOs more right space.

### 4.3. Analysis of public participation

(1) Analysis of the public participation degree q

The effect of public participation on the effectiveness of agricultural non-point source pollution governance is related to the value of the function *M*(*q*, *α*), letting the function *M*(*q*, *α*) derive from the public participation degree *q*.


$$\frac{\partial M(q,\alpha )}{\partial q}=-\varphi +\alpha \varphi +F-\lambda F$$ (23)

According to Eq (23), it can be seen that the position and negation of the equation is unknown, and it is related to the local government’s own investigation rate. Therefore, this paper discusses two different cases in terms of different government investigation rate.

1. when $\lambda <\frac{F-\varphi +\alpha \varphi }{F}$, then $\frac{\partial M(q,\alpha )}{\partial q}>0$. The function *M*(*q*, *α*) is positively correlated with the public participation degree *q*, increasing the value of *q* can make the function *M*(*q*, *α*) increase. Therefore, the public participation level needs to be increased to achieve better governance when government’s own investigation rate is low.
2. when $\lambda >\frac{F-\varphi +\alpha \varphi }{F}$, then $\frac{\partial M(q,\alpha )}{\partial q}<0$. the function *M*(*q*, *α*) is negatively correlated with the public participation degree *q*, decreasing the value of *q* can make the function *M*(*q*, *α*) increase. Therefore, the public participation level needs to be decreased to achieve better governance when government’s own investigation rate is high.

(2) Analysis of the Fairness of public reporting *α*

Ditto, letting the function M(q,α) derive from the public reporting fairness *α*.


$$\frac{\partial M(q,\varphi )}{\partial \alpha }=q\varphi >0$$ (24)

According to Eq (24), it can be seen that the function M(q,α) is an increasing function of the public reporting fairness *α*, then increasing the value of *α* can make the function M(q,α) increase. Therefore, in order to better satisfy the range of parameters for the evolution of the system towards the (1,1) point, the public reporting fairness needs to be increased.

## 5. Numerical simulation

In order to improve the efficiency of agricultural non-point source pollution management, the ideal situation of the evolutionary game system is to evolve towards the (1,1) point. Therefore, in this section, based on relevant literature and case experiences, the actual data of enterprises and government are assumed under the premise that the system evolves to the (1,1) point. In addition, the unit of measurement for all data is unified to one for the convenience of calculation. Assuming that the initial time of evolution is 0 and the end time is 15, the initial proportion of local government adopting strategy *x* among their groups is 0.5; meanwhile, the proportion of LPBE adopting strategy *y* among their groups is 0.5. Meanwhile, the initial values of other parameters are set as *s* = 8, *C*_0_ = 3, *p* = 0.9, *ω* = 0.7, *q* = 0.5, *α* = 0.8, *n* = 2, *R*_*e*_ = 3, *d* = 3, *φ* = 2, *F* = 3, *λ* = 0.9, *k* = 0.4.

### 5.1. Analysis of purifying subsidy

**Fig 2:**
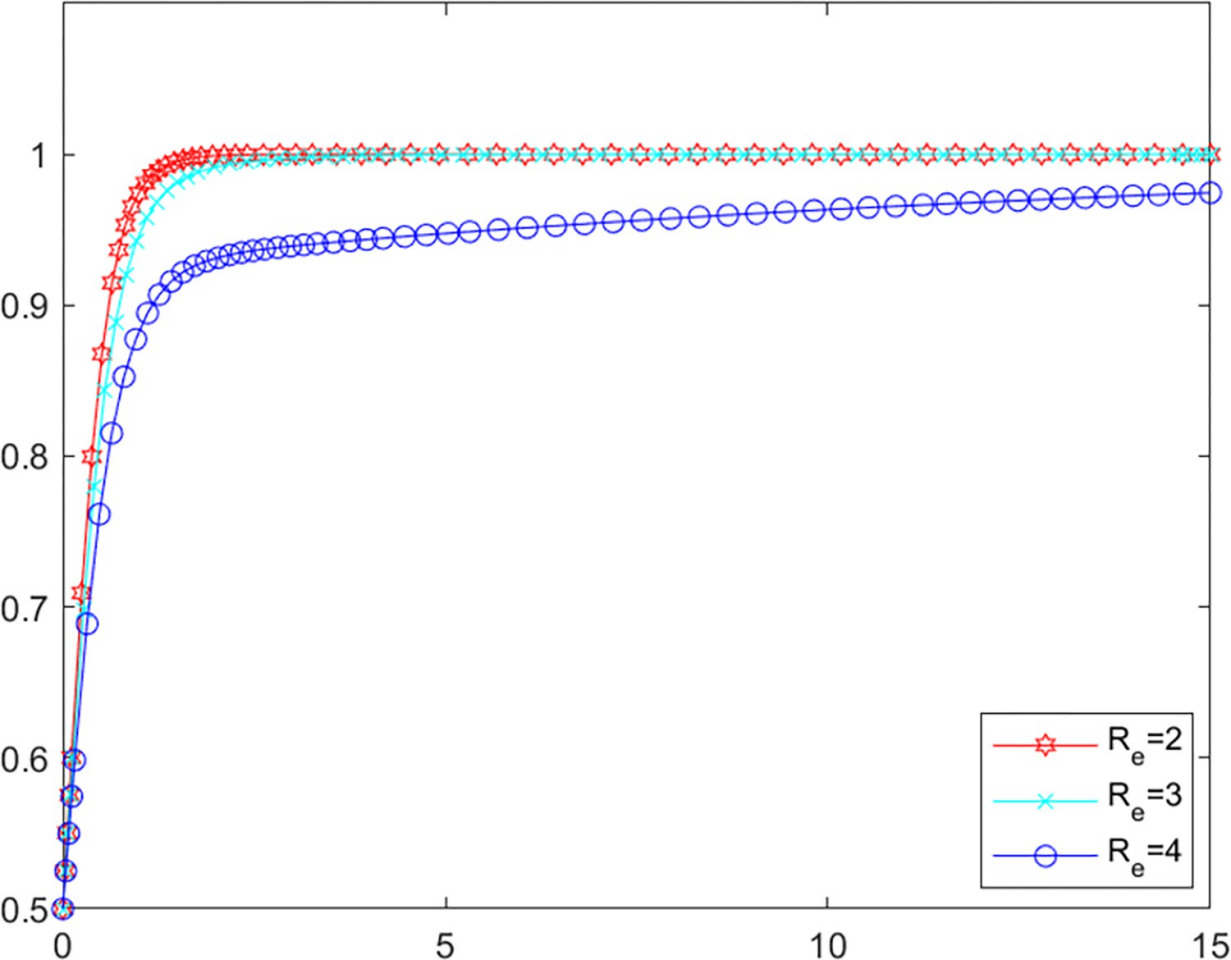
Government’s evolutionary path.

**Fig 3:**
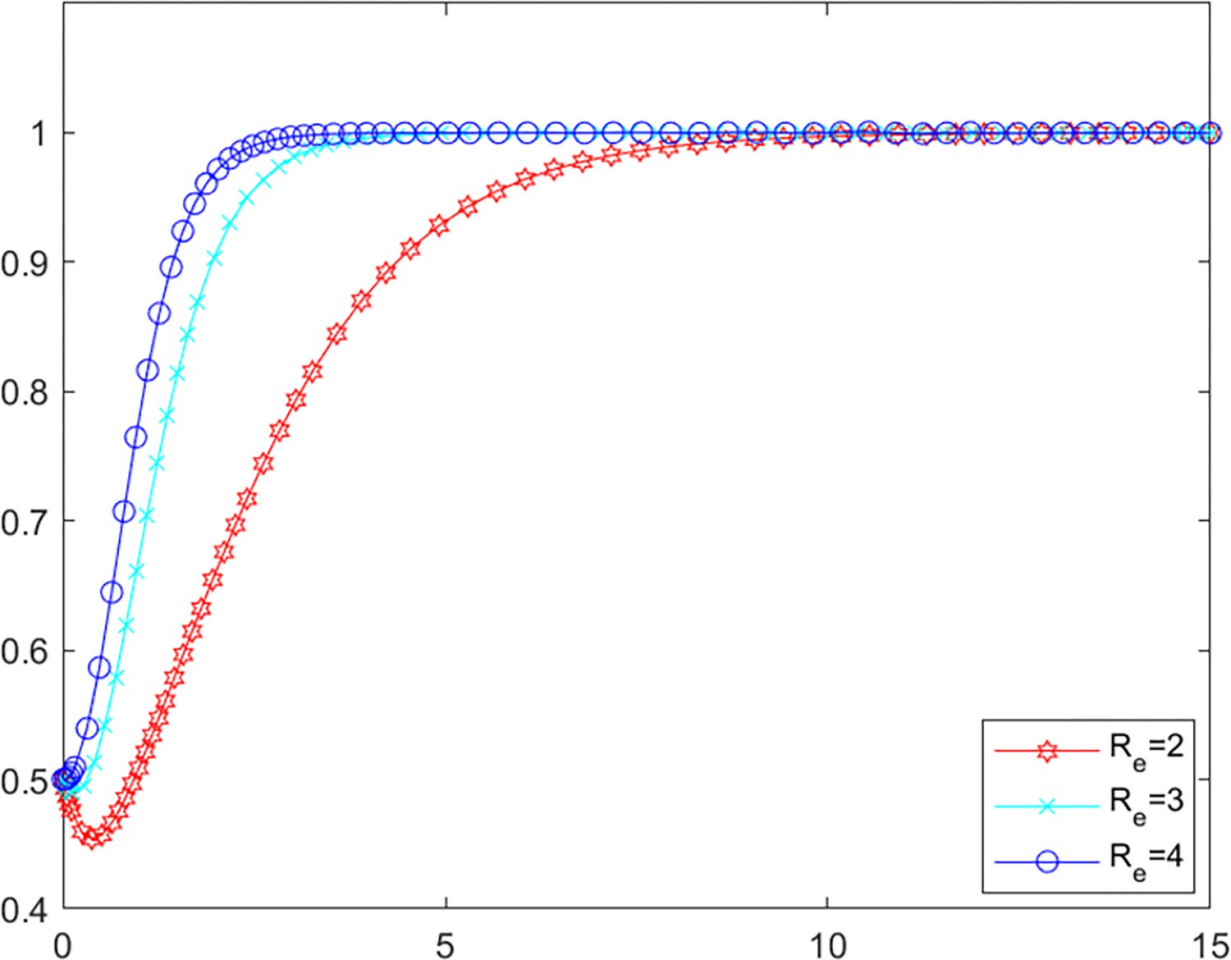
LPBE’s evolutionary path.

In order to investigate the effect of the purifying subsidy *R*_*e*_ on the evolutionary game system, this paper draws on the existing literature by assigning different values to the parameters instead of the policy intensity, i.e. the policy intensity increases as the value of the parameter increases [45, 46]. The initial values of the other parameters are kept constant, the values of *R*_*e*_ is assigned as 2, 3 and 4 respectively for simulation in MATLAB software. The evolutionary paths of local government as well as LPBE are shown in Figs 2 and 3.

Combining Figs 2 and 3, it can be seen that as the purifying subsidy increases, the local government reaches the evolutionary steady state more slowly, while LPBE arrives at this state more quickly, which indicates that purifying subsidy can be an effective incentive for LPBE to adopt purifying strategy. However, excessive purifying subsidies may burden government finances, and accordingly, the willingness of the government to adopt positive supervision strategy decreases. The subsidy has an incentive effect on LPBE [47], but the local government needs to choose different subsidy strategies depending on their circumstances.

### 5.2. Analysis of parameters for ENGOs

**Fig 4:**
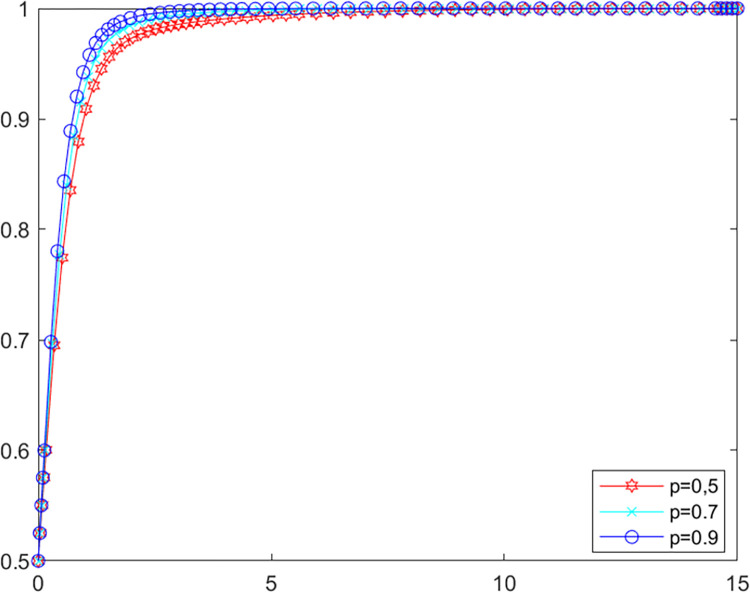
Government’s evolutionary path (p).

**Fig 5:**
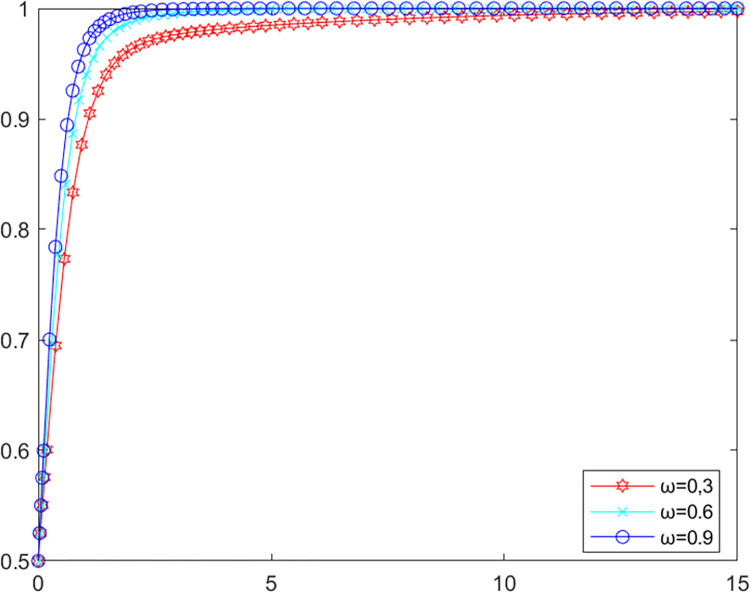
Government’s evolutionary path (*ω*).

In order to investigate the influence of the parameters related to ENGOs on the evolutionary game system, the initial values of the other parameters are kept constant, setting *p* as 0.5, 0.7, 0.9 and *ω* as 0.3, 0.6, 0.9 respectively into MATLAB software for simulation. The evolutionary path of the local government in the system is shown in Figs 4 and 5.

As shown in Figs 4 and 5, increasing ENGOs participation level and right space will encourage local government to reach an evolutionarily stable state more quickly. Previous studies have shown that local ENGOs play a crucial role in local environmental governance [48], they achieve environmental improvements through increased investment in environmental protection [49], but sufficient space for political opportunities is needed for them to mobilize and organize public participation in environmental governance [50]. Accordingly, to achieve better effects in the multiple governance of agricultural non-point source pollution, local government should encourage more ENGOs to participate in the governance of agricultural non-point source pollution as well as improve the right space of local ENGOs.

### 5.3. Analysis of parameters for public

#### (1) Public participation level influence

As the role of public participation in the multiple governance of agricultural non-point source pollution is influenced by the local government’s own investigation rate, whose threshold value is 0.87, without affecting the overall findings, the study of the public participation level is divided into the following two scenarios.

**Fig 6:**
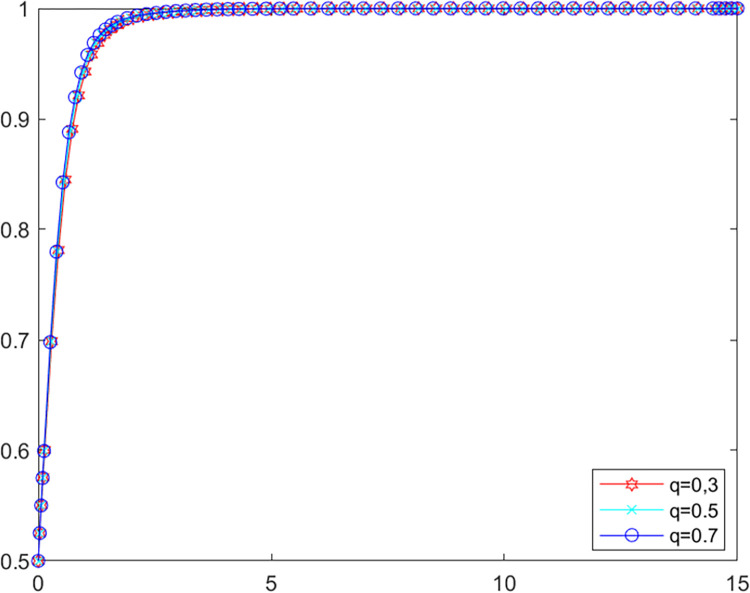
Government’s evolutionary path.

**Fig 7:**
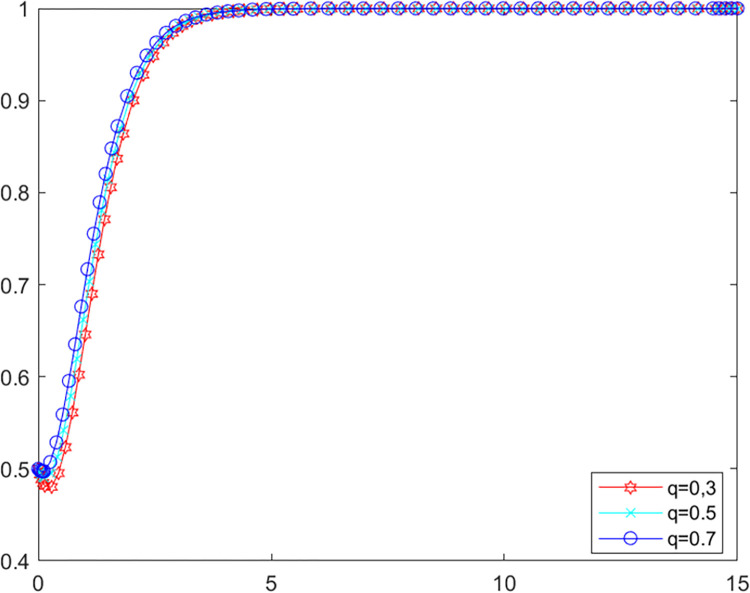
LPBE’s evolutionary path.

**Scenario one:** The local government’s own investigation rate is higher than 0.87. The initial values of other parameters are kept constant at this point, let *λ* = 0.9, set *q* as 0.3, 0.5 and 0.7 respectively into MATLAB software for simulation. The evolutionary paths of local government and LPBE in the system are shown in Figs 6 and 7.

**Fig 8:**
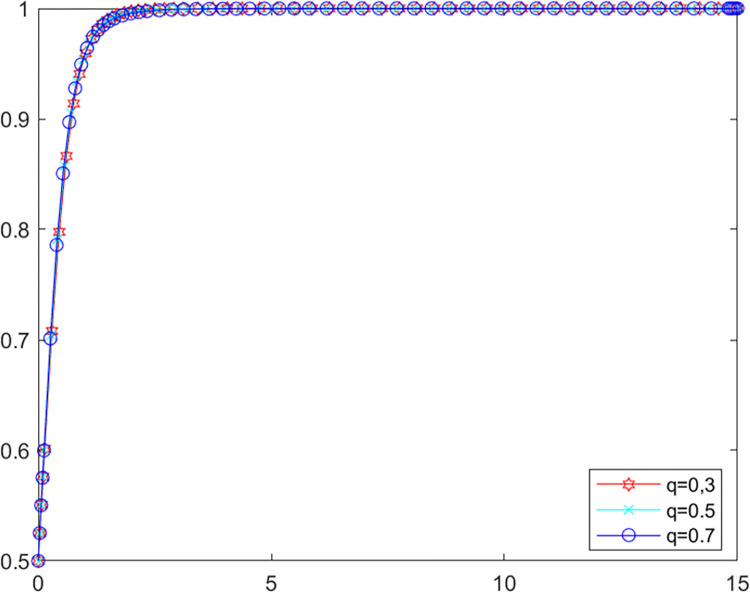
Government’s evolutionary path.

**Fig 9:**
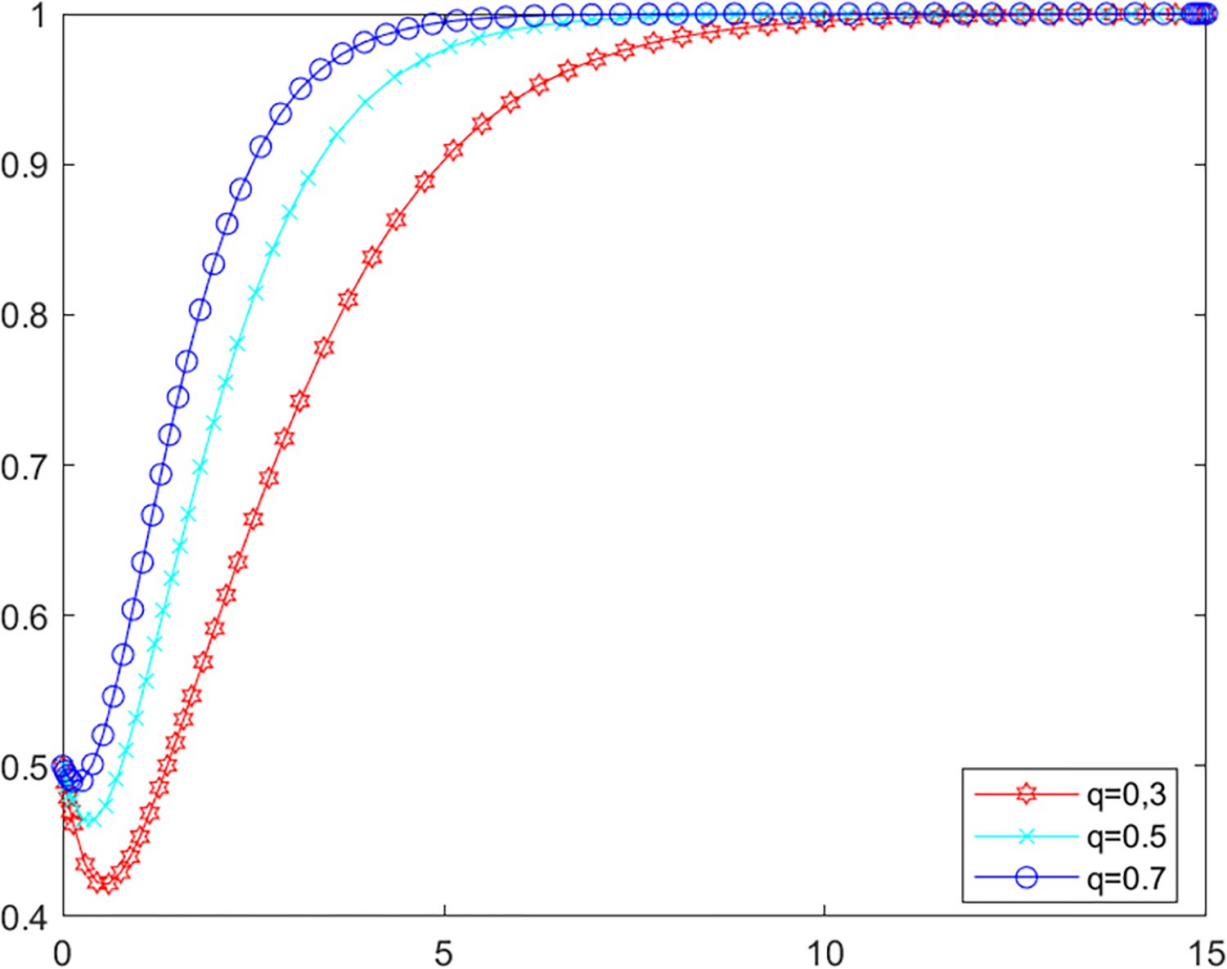
LPBE’s evolutionary path.

**Scenario two:** The local government’s own investigation rate is lower than 0.87. The initial values of other parameters are kept constant at this point, let *λ* = 0.4, set *q* as 0.3, 0.5 and 0.7 respectively into MATLAB software for simulation. The evolutionary paths of local government and LPBE in the system are shown in Figs 8 and 9.

Combining scenarios 1 and 2, it can be seen that when the local government’s own investigation rate is high, public participation has basically no influence on the strategy choice of the local government and LPBE. In this case, the local government can effectively control the emissions of LPBE by its supervision capacity alone. Conversely, when the local government’s own investigation rate is low, the local government cannot effectively control the emissions of LPBE by its supervision capacity alone, at which point public participation can, to a certain extent, replace the government’s supervision, thus playing a positive role in environmental governance [51]. As shown in Fig 6, increasing public participation can effectively promote LPBE to choose purifying blowdown strategy.

#### (2) Public reporting fairness influence

**Fig 10:**
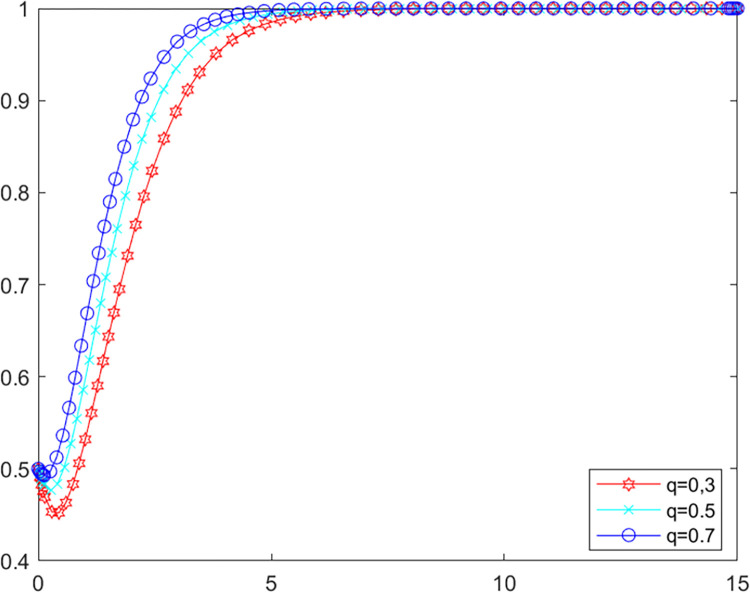
LPBE’s evolutionary path (*α* = 0.8).

**Fig 11:**
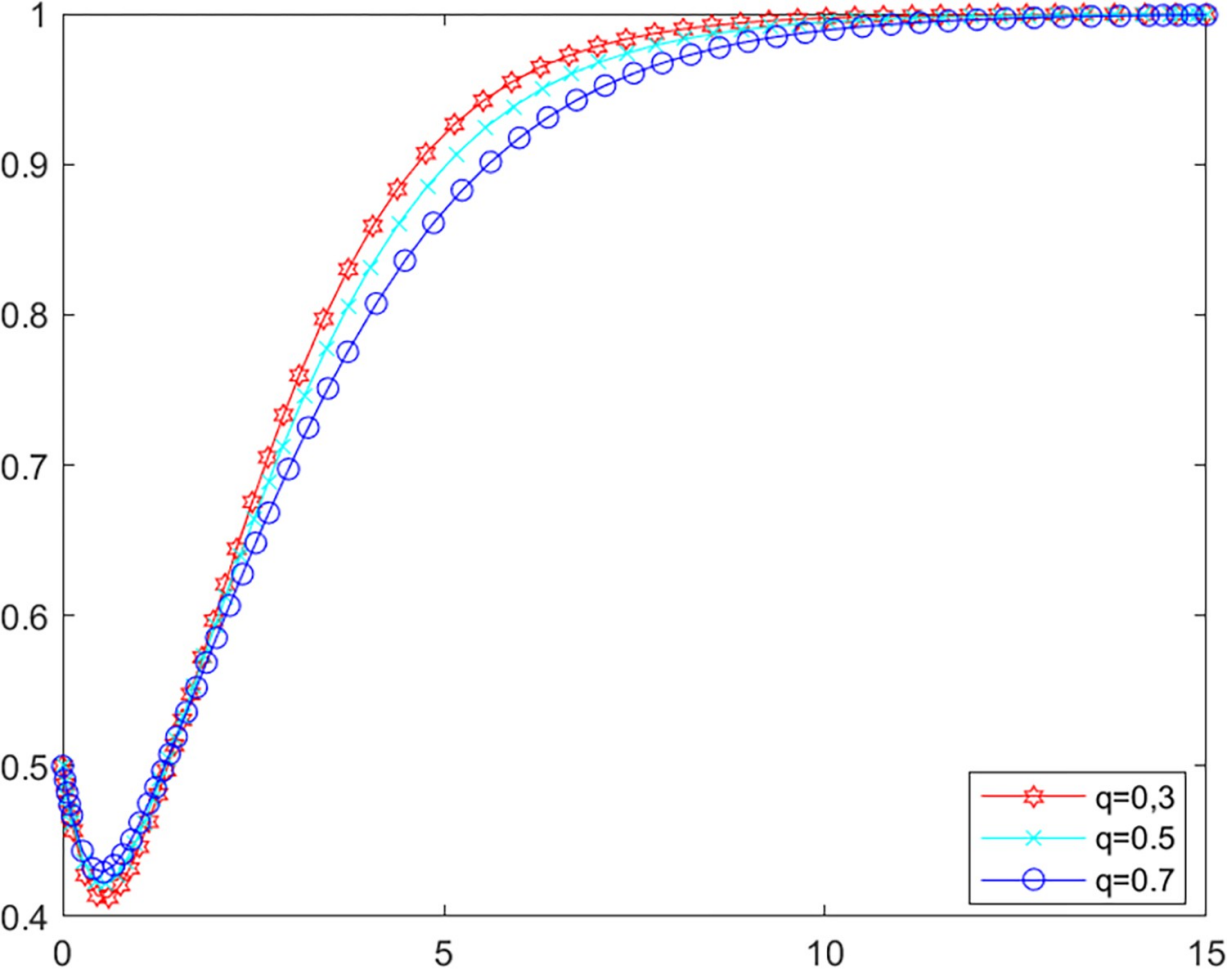
LPBE’s evolutionary path (*α* = 0.2).

Public participation in environmental behavior has a direct positive impact on regional environmental governance performance [52]. However, misguided public opinion and unfair reporting information bring additional losses to local government as well as LPBE. Above analysis shows that public participation has no effect on the game system when the government’s own investigation rate is higher than 0.87, so the following analysis assumes that *λ* = 0.6. In order to deeply explore the impact of public reporting fairness *α* on the evolutionary game system, assigning values of *α* to 0.8 and 0.2 and *q* to 0.3, 0.5 and 0.7 respectively to MATLAB software for simulation. The evolutionary path of LPBE in the system are shown in Figs 10 and 11.

As can be seen from Figs 10 and 11, public participation has a positive impact on LPBE’s choice of purifying strategy when public reporting fairness is high. Conversely, when public reporting fairness is low, public participation harms LPBE’s choice of purifying strategy. Comparing Figs 10 and 11, it can be seen that the rate at which LPBE reaches evolutionary stability decreases with the decrease in public reporting fairness, implying that increasing public reporting fairness improves the motivation of LBPEs to purify their emissions. The above findings suggest that public reporting fairness plays a crucial role in the multiple co-governance of agricultural non-point source pollution. In order to improve the efficiency of pollution governance, local government should not only improve the new media mechanism for public participation in environmental monitoring [53], but also focus on the effectiveness of public participation and the authenticity of relevant reporting information.

## 6. Conclusions

### 6.1. Research findings and implications

The local government’s implementation of multiple co-governance for agricultural non-point source pollution requires considering the participation of multiple factors such as ENGOs and the public in the governance process, and exploring the conditions under which each act effectively. This paper constructs an evolutionary game model of the multiple co-governance for agricultural non-point source pollution, so as to analyze the influence of related parameters on the stable evolutionary state of the system. conclusions are drawn as follows. Firstly, government subsidy is a double-edged sword, which promotes enterprises emission reduction while reducing the government’s regulatory willingness. Second, increasing the level of participation and rights space of ENGOs are conducive to improving the efficiency of agricultural surface source pollution control. Finally, with high effectiveness of public reporting, resident participation has an important supervisory role for enterprises and the government.

### 6.2. Policy recommendations

First of all, the magnitude of the government subsid should be determined according to government’s own actual situation. When local government finance is sluggish, reducing the amount of subsidy while increasing supervision has an indirect role on the governance. When local government is financially prosperous, increasing the amount of purifying subsidy has better effect on the governance.

Secondly, local government should improve the participation enthusiasm and right space of ENGOs, so as to achieve better pollution governance effects. On the one hand, the effectiveness of the interaction between government and ENGOs should be enhanced. The government needs to take the initiative to assume the primary responsibility of framing the governance model of government-society cooperation, and improve the government information disclosure mechanism while enriching the channels of institutional participation. At the same time, an environmental advisory committee with representatives from the government, ENGOs, and experts should be established to draw in social forces to participate in public affairs. On the other hand, the government should provide institutional support for ENGOs to express their environmental demands through market paths, create multifaceted incentive support for ENGOs that provide quality services, and supply corresponding technical, financial, and policy guarantees.

Finally, in order to make public participation play an active role, local government should strengthen science education and publicity on agricultural non-point source pollution control policies and activities to help residents understand how to participate in environmental protection. Encourage residents to participate in and organize activities, strengthen the exchange of environmental information, and increase the accumulation of social capital. Establish a public participation mechanism of "prior consultation, process participation and post-evaluation" to protect the public’s environmental rights and interests and strengthen the relationship and trust between the government and the public. Through special lectures, volunteer activities, and friendly family selection activities, residents’ participation in environmental information collection, environmental problem identification, and environmental management is enhanced.

### 6.3. Limitations and future research directions

There is still some important extension work to be carried out in the future, which are neglected for the sake of clarity and simplicity in this paper. Firstly, in order to simplify the model, this paper assumes that the relationship between ENGOs participation and government supervision cost is linear, though the actual situation may be more complicated. Secondly, the model supposes that the groups have bounded rationality, regardless of their social preferences. Both aspects deserve further research in the future.

## Supporting information

S1 Data (DOCX)
