# Supplementary material for: Research on multiple co-governance of agricultural non-point source pollution in China on the perspective of ENGOs and public participation
Source: PLoS One. 2023 Feb 9;18(2):e0280360. doi: 10.1371/journal.pone.0280360 (PMC9910745; doi:10.1371/journal.pone.0280360)
Supplement: S1 Data — (DOCX) [file pone.0280360.s001.docx]

| Parameters  Figure | $s$ | $C_{0}$ | $p$ | $\omega$ | $q$ | $\alpha$ | $n$ | $R_{e}$ | $d$ | $\varphi$ | $F$ | $\lambda$ | $k$ |
| --- | --- | --- | --- | --- | --- | --- | --- | --- | --- | --- | --- | --- | --- |
| 2 | 8 | 3 | 0.9 | 0.7 | 0.5 | 0.8 | 2 | 2 | 3 | 2 | 3 | 0.9 | 0.4 |
|  | 8 | 3 | 0.9 | 0.7 | 0.5 | 0.8 | 2 | 3 | 3 | 2 | 3 | 0.9 | 0.4 |
|  | 8 | 3 | 0.9 | 0.7 | 0.5 | 0.8 | 2 | 4 | 3 | 2 | 3 | 0.9 | 0.4 |
| 3 | 8 | 3 | 0.9 | 0.7 | 0.5 | 0.8 | 2 | 2 | 3 | 2 | 3 | 0.9 | 0.4 |
|  | 8 | 3 | 0.9 | 0.7 | 0.5 | 0.8 | 2 | 3 | 3 | 2 | 3 | 0.9 | 0.4 |
|  | 8 | 3 | 0.9 | 0.7 | 0.5 | 0.8 | 2 | 4 | 3 | 2 | 3 | 0.9 | 0.4 |
| 4 | 8 | 3 | 0.5 | 0.7 | 0.5 | 0.8 | 2 | 3 | 3 | 2 | 3 | 0.9 | 0.4 |
|  | 8 | 3 | 0.7 | 0.7 | 0.5 | 0.8 | 2 | 3 | 3 | 2 | 3 | 0.9 | 0.4 |
|  | 8 | 3 | 0.9 | 0.7 | 0.5 | 0.8 | 2 | 3 | 3 | 2 | 3 | 0.9 | 0.4 |
| 5 | 8 | 3 | 0.9 | 0.3 | 0.5 | 0.8 | 2 | 3 | 3 | 2 | 3 | 0.9 | 0.4 |
|  | 8 | 3 | 0.9 | 0.6 | 0.5 | 0.8 | 2 | 3 | 3 | 2 | 3 | 0.9 | 0.4 |
|  | 8 | 3 | 0.9 | 0.9 | 0.5 | 0.8 | 2 | 3 | 3 | 2 | 3 | 0.9 | 0.4 |
| 6 | 8 | 3 | 0.9 | 0.7 | 0.3 | 0.8 | 2 | 3 | 3 | 2 | 3 | 0.9 | 0.4 |
|  | 8 | 3 | 0.9 | 0.7 | 0.5 | 0.8 | 2 | 3 | 3 | 2 | 3 | 0.9 | 0.4 |
|  | 8 | 3 | 0.9 | 0.7 | 0.7 | 0.8 | 2 | 3 | 3 | 2 | 3 | 0.9 | 0.4 |
| 7 | 8 | 3 | 0.9 | 0.7 | 0.3 | 0.8 | 2 | 3 | 3 | 2 | 3 | 0.9 | 0.4 |
|  | 8 | 3 | 0.9 | 0.7 | 0.5 | 0.8 | 2 | 3 | 3 | 2 | 3 | 0.9 | 0.4 |
|  | 8 | 3 | 0.9 | 0.7 | 0.7 | 0.8 | 2 | 3 | 3 | 2 | 3 | 0.9 | 0.4 |
| 8 | 8 | 3 | 0.9 | 0.7 | 0.3 | 0.8 | 2 | 3 | 3 | 2 | 3 | 0.4 | 0.4 |
|  | 8 | 3 | 0.9 | 0.7 | 0.5 | 0.8 | 2 | 3 | 3 | 2 | 3 | 0.4 | 0.4 |
|  | 8 | 3 | 0.9 | 0.7 | 0.7 | 0.8 | 2 | 3 | 3 | 2 | 3 | 0.4 | 0.4 |
| 9 | 8 | 3 | 0.9 | 0.7 | 0.3 | 0.8 | 2 | 3 | 3 | 2 | 3 | 0.9 | 0.4 |
|  | 8 | 3 | 0.9 | 0.7 | 0.5 | 0.8 | 2 | 3 | 3 | 2 | 3 | 0.9 | 0.4 |
|  | 8 | 3 | 0.9 | 0.7 | 0.7 | 0.8 | 2 | 3 | 3 | 2 | 3 | 0.9 | 0.4 |
| 10 | 8 | 3 | 0.9 | 0.7 | 0.3 | 0.8 | 2 | 3 | 3 | 2 | 3 | 0.6 | 0.4 |
|  | 8 | 3 | 0.9 | 0.7 | 0.5 | 0.8 | 2 | 3 | 3 | 2 | 3 | 0.6 | 0.4 |
|  | 8 | 3 | 0.9 | 0.7 | 0.7 | 0.8 | 2 | 3 | 3 | 2 | 3 | 0.6 | 0.4 |
| 11 | 8 | 3 | 0.9 | 0.7 | 0.3 | 0.2 | 2 | 3 | 3 | 2 | 3 | 0.6 | 0.4 |
|  | 8 | 3 | 0.9 | 0.7 | 0.5 | 0.2 | 2 | 3 | 3 | 2 | 3 | 0.6 | 0.4 |
|  | 8 | 3 | 0.9 | 0.7 | 0.7 | 0.2 | 2 | 3 | 3 | 2 | 3 | 0.6 | 0.4 |
